# Innovational combination of hetero-bifunctional N-PEG quinoline scaffolds derivatives with improved anticancer activity against breast and colon cancer cell lines and P-glycoprotein, cytochrome p450 enzyme activity prediction

**DOI:** 10.3906/kim-2006-13

**Published:** 2020-12-16

**Authors:** Mahesh GAIDHANE, Ajay GHATOLE, Kushal LANJEWAR, Kishor HATZADE

**Affiliations:** 1 Department of Chemistry, Shri Lemdeo Patil Mahavidyalaya, RTMNU, Mandal, MS India; 2 Department of Chemistry, Dhote Bandhu Science College, RTMNU, Gondia, MS India; 3 Department of Chemistry, Mohsinbhai Zaweri Mahavidyalaya, Desaiganj, Wadsa, MS India

**Keywords:** PEGylated quinoline, breast, colon cancer, P-glycoprotein, cytochrome P450 enzyme

## Abstract

Polyethylene glycol (PEG) is a polymer that is widely used as a carrier for drug delivery systems (DDS). A library of N-PEGylated quinoline derivatives of PEG molecular weight 200 was prepared rapidly after the activation of PEGs using maleic anhydride. Quinoline with a polymer backbone is essential as new material. PEG is a water-soluble nonionic polymer approved by food and drug organizations for medicine applications. Because of its nontoxic grapheme, it is widely utilized in numerous biochemical, cosmetic, pharmaceutical, and industrialized applications. The modern SwissADME is a web tool that stretches free admittance to a pool of hasty, yet solid, clarifying models for physicochemical properties, pharmacokinetics, and therapeutic science. The present facile synthetic strategy can be a practical approach for incorporating polymeric carriers conjugated with drug moieties, either in the backbone of the polymer or as a terminal and pendant group on the polymer chains.

## 1. Introduction

A library of N-polyethylene glycol(PEG)ylated quinoline derivatives of PEG molecular weight 200 was prepared rapidly after the activation of PEGs using maleic anhydrides. Quinoline with a polymer backbone is essential as new material. In 1995, Zalipskyet al.[1] and Herman et al. [2] reported the functionalization of polyethylene glycol for the preparation of biologically-relevant conjugates along with some reactive end groups. Polyethylene glycol conjugation chemistry represents an emerging trend for the generation of potential therapeutic agents. Woodle et al. [3] and Allen et al. [4] have demonstrated that PEG-modified biological molecules can benefit from extended plasma lifetimes, induced by reducing the uptake by the reticuloendothelial system and more generally, from a decrease of the undesired consequences of electrostatic and van der Waals interactions. A future direction towards nonviral gene therapy is the use of PEG-grafted synthetic vectors as long-circulating carriers for receptor-mediated gene delivery [5]. PEG has been used as a solvent medium for regioselective Heck reaction with elementary recyclability of solvents [6]. The incomparable ability of PEG to be soluble in both aqueous solution and organic solvents makes it eligible for end grouping derivatization and chemical conjugation to biological molecules under insignificant physiological conditions. The PEG-modified drug has been used widely as an antitumor drug carrier because of its excellent water solubility and biocompatibility [7]. Norfloxacin, one of the fluoroquinolone antibiotics, was conjugated to mannosylated dextrin to increase the intake of the drug by cells, enabling faster access to microorganisms, [8,9]. The backbone of polyurethanes and other ordinary low-density polyethylenes with antimicrobial Norfloxacin drugs were investigated by Yang et al. [10] against several gram-positive, and gram-negative bacteria, and displayed excellent antimicrobial activities. Recent advances in tumor therapy has demonstrated that successful anticancer strategies could be developed by employing proper carrier systems able to deliver probes, drugs, or genes to tumors targets [11]. Quinoline derivatives have been widely used for their biological [12–14], antibacterial, and antimalarial actions and in addition, for their cardiovascular, antineoplastic, and receptor agonist actions [15]. PEG, a water-soluble, nonionic polymer, which has a nontoxic character, is widely used as a carrier for drug delivery systems, and in many biochemical, cosmetic, pharmaceutical, and business applications [16]. The novel facile synthesis of a family of N-PEGylated quinoline by coupling PEG _200(n)_ with 2-formyl, 3-chloro, 6-amino quinoline via maleic anhydride as an activator was presented herein. Antimicrobial activities of the quinoline-PEG polymer and quinoline were tested against gram-positive and gram-negative bacteria. The results indicated that the new polymers had potential as potent antimicrobial agents. Since these polymers were comparatively stable at high temperatures, they can be used for medical and biomaterial applications with prior thermal sterilization. SwissADME permits the review of absorption, distribution, metabolism, and excretion (ADME) restrictions of medication aspirants and small molecules and provides statistics that authorize the early hazard calculation in drug enhancement progression. Exceptionally, SwissADME affords a point to estimate Lipinski’s rule of five [17] for medication semblance of oral bioavailability. The present facile synthetic strategy can be a practical approach for incorporating the polymeric carriers conjugated with drug moieties, either in the backbone of the polymer or as a terminal and pendant group on the polymer chains.

PEG is the covalent link of nonpoisonous, hydrophilic polyethylene glycol (PEG) to dynamic pharmaceutical constituents [18]. The technology established from revolutionary work was conducted in the 1950s and 1960s on the coupling of polymers to protein. The amendment of a biopharmaceutical with polyethylene glycol increases its hydrodynamic area and reduces its immunogenicity and antigenicity. Other achievements included braked renal evacuation, enhanced stability in the direction of proteolysis, and increased solubility of the biopharmaceutical in aqueous solution [19]. The synthesis of macromolecules with different functional groups in 2 ends is a promising field of research. A lot of useful materials can be designed from the macromolecules. However, due to the difficulty of separation of the reacted mixture, significant achievements have not yet been made. Pozzo et al. reported that some monofunctional derivatives of PEG could be prepared and separated using excess PEG, but the molecular weight of the monosubstituted PEGs was no more than 1000. It was evident that the higher the molecular weight of the starting PEG, the more difficult it was for the isolation of monosubstituted PEG from the reacted mixture. This is because there was no sharp difference between monosubstituted, disubstituted, and raw PEGs in the chemical and physical properties [20–22].Heterocyclic mixes have increased gigantic significance as a result of their low reverberation vitality and solid hydrogen‐bonding capacities [23]. A recent investigation reported the fruitful synthesis of new heterocyclic-like ester and amide under water as a solvent and the surface of silica-coated CoFe2O4 magnetic nanoparticles for the synthesis of 3-pyrrolin-2-ones [24], as well as for the preparation of 2-amino-4,6-diarylnicotinonitrile under microwave irradiation [25].

## 2. Result and discussion

An extension of PEG-supported chemistry was linked in the arena of functionalized polymers as reagent supports. Prepared was novel PEG N-terminal quinoline substituted derivatives and it was shown that, when related to a heterogeneous commercially-existing counterpart, they held promising reaction kinetics equal to the Staudinger and Mitsunobu etherification reactions. They were evaluated for their in vitro anticancer, antibacterial, and antifungal activities. The present study expanded on those preliminary investigations to explore the synergistic effects of such pharmacophores.

The PEGylation chemistry successfully demonstrated at the amino group was situated at C-7 of the quinoline hybrids. To couple a PEG to a quinoline molecule, it is first necessary to activate the polymer by converting the hydroxyl terminus to some functional group proficient reacting with the functional group situated on quinoline molecule. Herein, it was opted to activate the PEG by turning it to its acid derivative and coupling it with an amino group of quinoline molecules. This PEGylation methodology involved the conjugation of quinoline onto hetero-bifunctional PEG derivatives that had -OH on one terminus and -COOH on the other for covalent attachment of the amino group. The free carboxyl group of compounds was used for conjugation through an amine connection. It was determined that this conjugation proceeded through the formation of amide linkage between the PEG chain and quinoline under mild reaction conditions.

In its most common form, PEG is a linear or branched polyether terminated with hydroxyl groups and has the general structure as shown below:

HO-(CH _2_ CH _2_ O) _n_ -CH _2_ CH _2_ -OH.

PEG is synthesized by anionic ring-opening polymerization of ethylene oxide initiated by the nucleophilic attack of a hydroxide ring.

PEG _200(n)_ (5 mL, 0.028 moles, 200 g/mole) was dissolved in 20 mL of dry CH _2_ Cl _2_ . Added to this solution was tetrahydrofuran (THF) containing maleic anhydride (0.056 mol, 0.54 mg) and pyridine (0.056 mol, 0.46 mL). After stirring overnight at room temperature, the reaction was completed, as indicated by thin-layer chromatography (TLC) (methanol:ethyl acetate, 7:3) (no anhydride left and the detection of a single polar PEG compound). The monoacid derivative of the poly (ethylene glycol)200(n) was used without purification. The most useful for polypeptide modification is monoacid PEG, which has the general structure:

HO-(CH _2_ CH _2_ O) n-CH _2_ CH _2_ -O-COOH.

Now, for this monoacid derivative of PEG coupled to a 6-amino-2-chloroquinoline-3-carbonitrile (3a), it was necessary to activate the PEG by maleic anhydride. In the present study, the solution synthesis of a novel family of PEGylating reagent was presented, which contained a free -COOH terminus. Conditions were developed for the coupling of this novel PEGylating reagents to the -NH _2_ function of model quinoline to illustrate their general utility. Together, this study demonstrated that these PEGylating reagents were well-suited for conjugation peptide and protein containers, either free -COOH or -NH _2_ functions. These reagents might have wide utility in therapeutic expansion, as branched PEGylation has been observed to deliver more effective protection of proteins from proteolysis by protecting the protein surface approaching the macromolecules.

The synthesis of quinoline compounds 3a–3i (Scheme) was accomplished, starting with 6-amino-2-chloroquinoline-3-carbaldehyde (2). The treatment of compound 2 with ceric ammonium nitrate (CAN) in 30% aq. ammonia at 0–2 °C with constant stirring for 2–3 h gave 6-amino-2-chloroquinoline-3-carbonitrile (3a). However, the reaction of compound 2 with fused sodium sulfide in dry dimethylformamide (DMF), followed by hydroxyl amination, afforded 6-amino-2-mercaptoquinoline-3-carbaldehydeoxime (3b). Next, 3-(6-amino-2-chloroquinoline-3-yl)-1-p-tolylprop-2-en-1-one (3c) was prepared by the reaction of compound 2 with 4-methyl acetophenone in 40% ethanolic NaOH. Moreover, 4-(2-(6-amino-2-chloroquinoline-3-yl) vinyl phenol (3d) was synthesized by treating a mixture of compound 2 and p-cresol in AcOH/Ac2O under reflux for 6–7h. Additionally, compound 2 was treated with thiourea and urea in AcOH under reflux for 15–16h and produced 7-aminopyrimido(4,5-b)quinoline-2(1H)thione(3e) and 7-aminopyrimido(4,5-b)quinoline-2(1H)one (3f), respectively. Furthermore, 3-(P-tolylimino) methyl-2-chloroquinoline-6 amine (3g) was prepared by condensation of compound 2 with p-toludiene in acidic methanol. A noteworthy ring annulation was observed when compound 2 was condensed with hydrazine hydrate in ethanol under reflux for 5–6h, which afforded 1-H-pyrazolo (3 and 4b), and quinolin-6-amine (3h). The synthesis of (Z)-ethyl 3-(6 amino-2-chloroquinoline-3-yl)-2-cyanoacrylate (3i) was accomplished by condensation of compound 2 with ethyl cyanoacetate (Table 1).

**Scheme:**
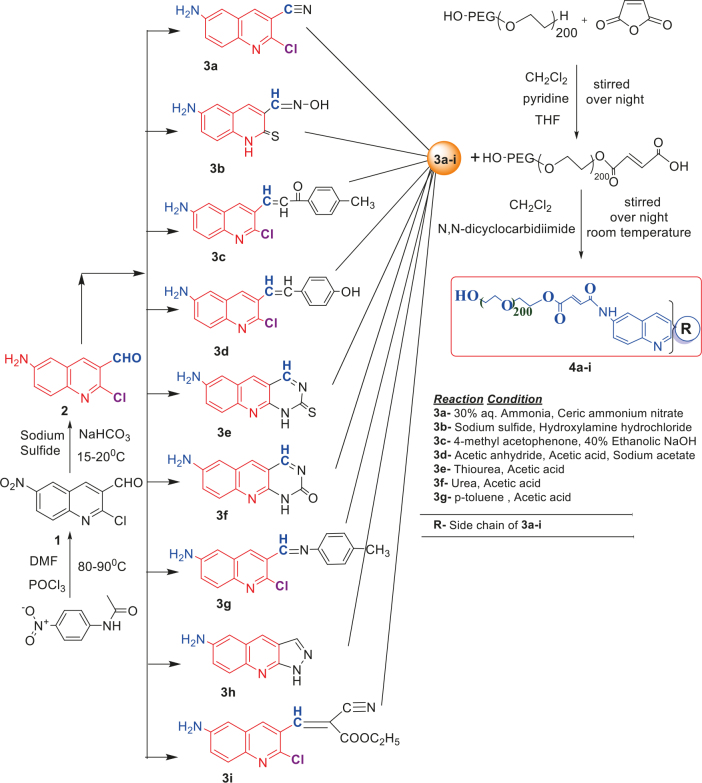
Hetero-bifunctional N-PEG quinoline scaffold derivatives.

**Table 1:** Physical characterization data of substituted quinoline derivatives 1, 2, and 3a–3i.

Entry	Product	Temperature	Yield (%)	m.p. (°C)	Molecularformula
1	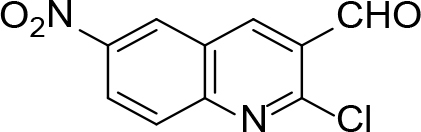	90 °C Heating	86	260	C10H5ClN2O3
2	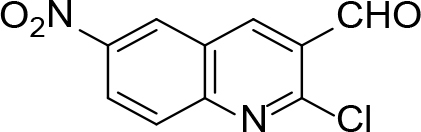	Refluxed	82	208	C10H7ClN2O
3a	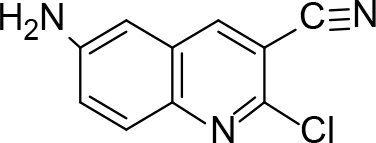	Stirring/RT	83	162	C10H6ClN3
3b	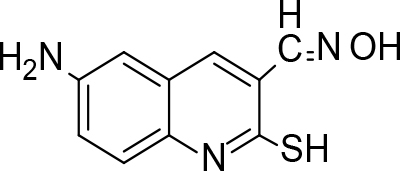	Stirring/RT	78	168	C10H9N3OS
3c	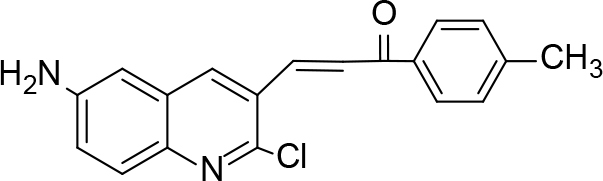	Stirring/RT	84	240	C19H15ClN2O
3d	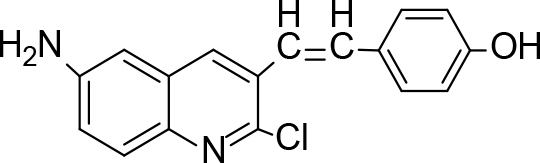	Refluxed	83	168	C17H13ClN2O
3e	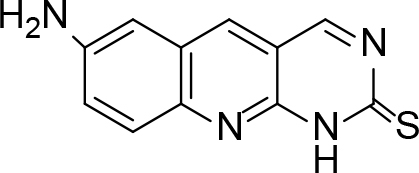	Refluxed	82	272	C11H8N4S
3f	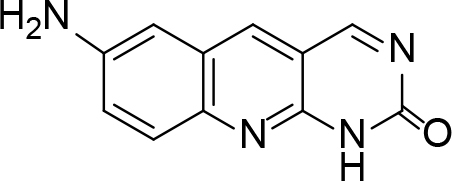	Refluxed	78	247	C11H8N4O
3g	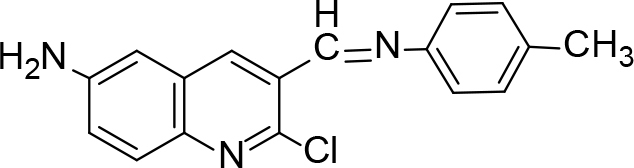	Refluxed	86	176	C17H14ClN3
3h	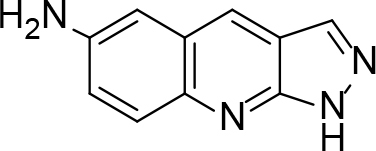	Refluxed	76	288	C10H8N4
3i	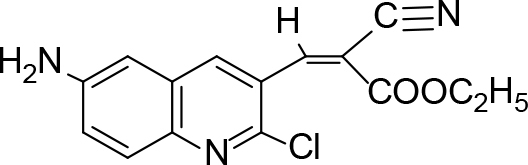	Refluxed	86	122	C15H12ClN3O2

The monoacid derivative of hydroxycarboxy PEG (HO-PEG200COOH) (28.0mmol) was activated with a 1:2molar equivalent of 6-amino-2-chloroquinolin-3-carbonitrile (2a) (46.0mmol) and N, N dicyclocarbidiimide (46.0mmol) dissolved in dichloromethane. The solution was stirred for 24 h at room temperature. A syrupy resin was obtained and dried under a vacuum. The syrupy resin was dissolved in 15 mL of acetone. A white precipitate of dicyclohexylurea (DCU) appeared and was discarded, and the filtrate was collected. The final filtrate was evaporated to afford the product. TLC (methanol:ethyl acetate, 7:3) was performed to check the existence ofDCU, which exposed a negative outcome that was confirmed by the infrared spectroscopy (IR) spectra. A negative dye test indicated complete amino group capping.

The resin was dehydrated in a vacuum for IR, 1HNMR, 13CNMR, and mass spectral analysis. At this point, the adhesive did not stick to the glass wall anymore. The IR spectrum of the resin showed the characteristic absorption band of the PEG ether backbone (1120 cm–1) and absorption bands (1760 and 1685 cm–1)for the ester and amide bond, respectively (C=O) (Table 2).

**Table 2:** tructures of the PEGylated quinolines (4a–4i).

S. No.	Structures of the PEGylated quinolines	Density (d) in cm3
4a	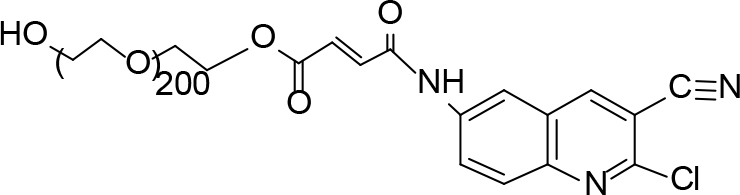	1.012
4b	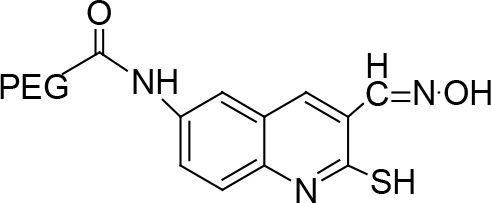	1.126
4c	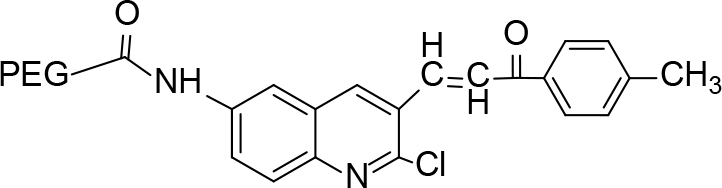	1.058
4d	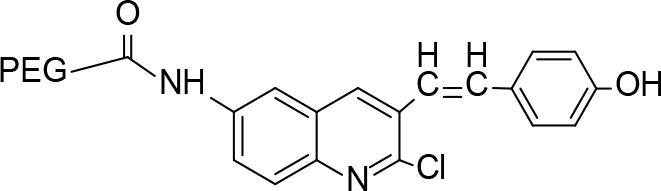	1.158
4e	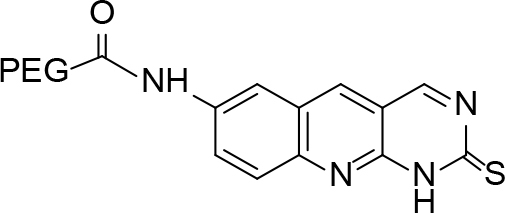	1.038
4f	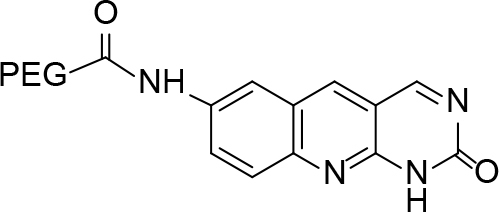	1.038
4g	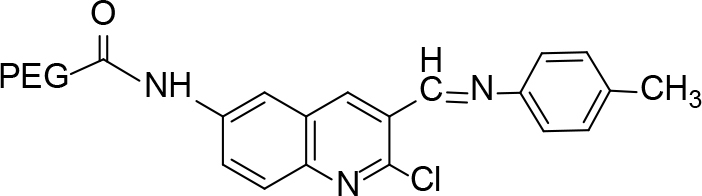	1.152
4h	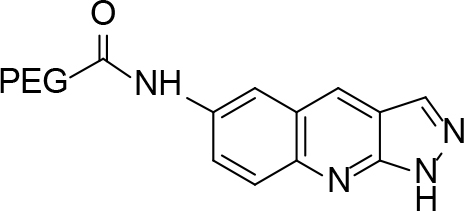	1.008
4i	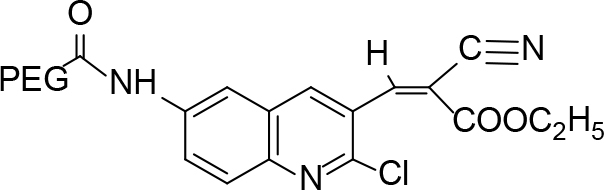	1.102

## 3. Experimental

All of the recorded melting points were taken in open capillary tubes and were uncorrected. The IR spectra were recorded with a FTIR PerkinElmer Rx-I instrument (PerkinElmer, Inc., Waltham, MA, USA) using Nujol.1HNMR spectra were recorded on 400 MHz Bruker AC-300 F instrument (Bruker BioSpin Corp., Billerica, MA, USA) using tetramethylsilane as the internal standard and deuterated chloroform as the solvent, and the mass spectra were recorded on a VG.70 S instrument at SAIF Chandigarh. The TLC technique was used to check the purity of the compound using silica gel G.

### 3.1. Biological assay

#### 3.1.1. In vitro anticancer assays

A library of 24 compounds was tested using the Alamar blue (AB) assay (in the case of leukemia cells) for their antiproliferative activity in vitro against 2 cancer cell lines: SW620, MD468, and MD468-2 were all colon cancer cell lines obtained from the American Tissue Culture Collection (ATCC, Manassas, VA, USA). MD468 is a mismatched repair-deficient cell line, and MD468-2 has a p53 mutation. The results of the cytotoxic activity in vitro were expressed as an ID50 (nM/mL), i.e. the concentration of the compound that inhibited the proliferation of 50% of the tumor cells when compared to the untreated control cells. Camptothecin (10 nM), Paclitaxel (10 nM), Doxorubicin (250 nM), and Oxaliplatin (2000 nM) were applied as referential cytotoxic agents (positive test controls). A value of less than 10 nM/mL was considered as an antiproliferative activity criterion for synthetic compounds. The results of the cytotoxicity studies are summarized in Table 3.

**Table 3:** Comparative IC50 nM of compounds 3a–3i and 4a–4i.

SrNo.	MDA MD 468 (breast cancer cell line)					SW480 (colon cancer cell)				
	Before PEGylating(3a–3i)		AfterPEGylating(4a–4i)		Improvement in efficacy (fold)	Before PEGylating(3a–3i)		AfterPEGylating(4a–4i)		Improvement in efficacy (fold)
1	17514	3a	37.3	4a	469	23839	3a	34.9	4a	682
2	77690	3b	22.6	4b	3430	98317	3b	39.3	4b	2499
3	12478	3c	20.8	4c	600	9077	3c	33.0	4c	275
4	82492	3d	28.2	4d	2921	72731	3d	24.1	4d	3014
5	97473	3e	39.5	4e	2469	83269	3e	29.5	4e	2823
6	102107	3f	20.9	4f	4876	108894	3f	33.0	4f	3297
7	102593	3g	49.8	4g	2061	135575	3g	19.1	4g	7114
8	100639	3h	8.5	4h	11896	86233	3h	9.3	4h	9256
9	20253	3i	7.8	4i	2607	56723	3i	27.4	4i	2068

IC50 values of the anticancer agents (controls).

#### 3.1.2.Antiproliferative activity

All 24 compounds were tested via AB assay (in the case of leukemia cells) for their antiproliferative activity in vitro against 2 cancer cell lines: SW620, MD468, and MD468-2 were all colon cancer cell lines obtained from the ATCC. MD468 is a mismatched repair-deficient cell line, and MD468-2 has a p53 mutation. The results of the cytotoxic activity in vitro were expressed as an ID50 (nM/mL), as stated above. Camptothecin (10nM), Paclitaxel (10 nM), Doxorubicin (250 nM), and Oxaliplatin (2000 nM) were applied as referential cytotoxic agents (positive test controls). A value of less than 10 nM/mL was considered as an antiproliferative activity criterion for synthetic compounds. The results of the cytotoxicity studies are summarized in Table 3.

In general, compounds 3a–4i did not display any anticancer activity, whereas compounds 4a–4i exhibited potent antiproliferative activity against theSW620, MD468, and MD468-2 colon cancer cell lines. This implied that after PEGylation, the anticancer activity enormously increased in multiples of 469- to 11896-fold. PEGylated 1-H-pyrazol (3 and 4b) and quinolin-6-amine (4h) exhibited the highest activity against the SW620, MD468, and MD468-2 colon cancer cell lines and had IC50 values of 8.5 nM/mL and 9.3 nM/mL, respectively, for the corresponding cell lines. Compound 4i had an IC50 value of 7.8 nM/mL and also possessed the highest anticancer activity against the SW620 colon cancer cell line. Compounds 4g and 4b showed lower activity among compounds 4a–4i against MD468, MD468-2, and SW620, respectively, with IC50 values of 49.8 nM/mL and 39.3 nM/mL. However, compounds 4a–4f displayed moderate activity when the IC50 values of all of the compounds were compared with those of standard drugs like Camptothecin (IC50:10 nM) and Paclitaxel (IC50: 10 nM). Doxorubicin (IC50: 250 nM) and Oxaliplatin (IC50: 2000 nM) were also suited as reference cytotoxic agents. A comparative investigation of the IC50 concentrations of compounds 4a–4i with those of Doxorubicin (IC50: 250nM) and Oxaliplatin (IC50: 2000nM) was conducted. The anticancer activity displayed at an IC50 concentration ranging from 7.8 nM/mL to 49.8 nM/mL indicated that all of the synthesized compounds exhibited incredibly higher potency than those of Doxorubicin (IC50: 250 nM) and Oxaliplatin (IC50: 2000 nM). The results summarized in Table 3 suggested that the potency of each compound as an anticancer agent was irrespective of its substituents and showed high selectivity and more specificity towards the binding targeted sites. Compound 4h proved to be a highly-potent candidate against the SW620, MD468, and MD468-2 colon cancer cell lines than the existing armory of anticancer drugs attributable to the ring annulations with fused pyrazole nucleus.

#### 3.1.3. Assay principle

In this assay, the viability of chemically-treated cells were measured by a dye, Resazurin or Alamar blue (AB). AB, a nonfluorescent pointer dye, changed to bright red-fluorescent resorufin through the reduction reactions of metabolically active cells. The quantity of fluorescence produced was directly proportional to the number of living cells.

Cells: For the initial screen of A549, the lung cancer cells were used. Positive hits were then used to screen 7 other cell lines used in the NIH assays.

**Figure 1:**
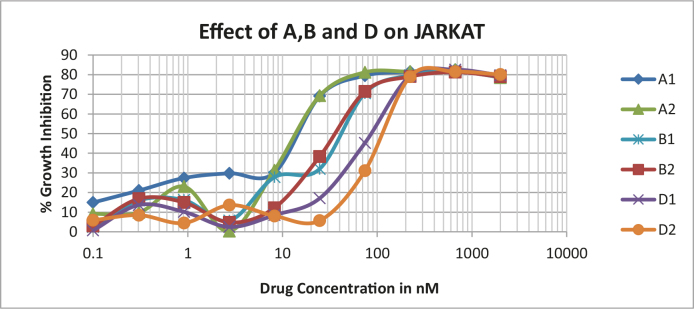
Effect of compounds 4a–4i in colon cancer line (SW620).

**Figure 2:**
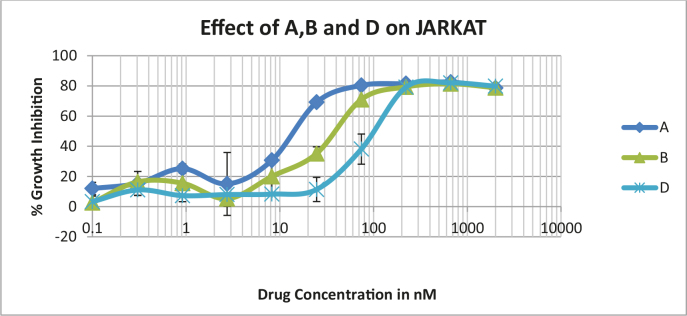
Effect of control standard anticancer drugs in SW620 cells.

Protocol: Standard AB assay protocol for cell viability assay was used. Confluently-grown cells were trypsinzed, and around 10,000 cells were seeded in each well of a 96-well plate. The next day, the medium was replaced with medium containing the appropriate concentration of the drug. The maximum concentration of each drug was 400µg/mL, and a one-quarter dilution was used for each drug. The cells were treated for 48 h and 2µL of AB was added to each well and incubated for 2 h. Fluorescence intensity was read with a plate reader. From the graph, the IC50 value was calculated. SW620, MD468, and MD468-2 were all colon cancer cell lines obtained from the ATCC. MD468 is a mismatched repair-deficient cell line, and MD468-2 has a p53 mutation. The effects of all of the new compounds on colon cancer cell lines SW620, MD468, and MD468-2, were observed and the results are depicted in Figures 1and 2. The growth inhibition percentages were compared with those of standard drugs Camptothecin, Paclitaxel, Doxorubicin, and Oxaliplatin (Table 3, Figures 1 and 2). For the rows, alcohol was added to generate killed cells.

#### 3.1.4. In vitro antibacterial and antifungal activity

All of the newly synthesized compounds were screenedin vitrofor their antimicrobial activity and antifungal activity against a variety of bacterial and fungal strains, such as *Staphylococcus Aureus* (gram +ve), *Escherichia Coli* (gram –ve), *Pseudomonas Aeruginosa* (Gram –ve), and *Candida Albicans* using the disc diffusion method. The nutrient agar broth was prepared by aseptic inoculation with 0.5 mL of 24-h-old subcultures of *S. Aureus* , *E. Coli* , and *C. Albicans* in separate flasks at 40–50 °C and mixing well by gentle shaking.

About 25 mL of the contents of the flask were poured and evenly spread in a Petri dish (13 cm in diameter) and allowed to set for 2h. Each test compound (20 mg) was dissolved in 2 mL of dimethyl sulfoxide (DMSO), which was used as a sample solution. A concentrated (100 µg/mL) solution was prepared using the dilution method. The sample size for all of the compounds was fixed as 10 µL.

The plates were incubated at 37 °C for 24 h, the control was similarly maintained with 1 mL of DMSO, and the zones of inhibition of the bacterial growth were measured in mm using a zone reader.

#### 3.1.5. In vitro antibacterial and antifungal activity

The newly synthesized compounds (3a–3i and 4a–4i) were tested for theirin vitro antimicrobial activity against clinical isolates of gram-positive bacteria S.Aureus, gram-negative bacteria E.coli and P. Aeruginosa, and the fungus C. Albicans. For all of the compounds, along with the standards for the bacteria, doxycycline, ampicillin, and fungi, fluconazole was used at a concentration of 100 µg/mL in DMSO as the solvent control and nutrient agar was used as the culture method [26–29]. After 24h of incubation at 37 °C, the zones of inhibition were measured in mm.

All of the synthesized compounds exhibited good to moderate activity against all of the microbes. No zone of inhibition was observed against the strains of *P. Aeruginosa* with compounds 3c, 3h, and 4c, while all of the compounds displayed significant activity against the strains of C. Albicans, E. Coli, and S. Aureus.

The toxicity increased with the increase in the concentration of the test solution containing new compounds, suggesting a maximum tolerated dose. Some compounds did not meet the conventional bacteriostatic standards at lower concentration levels. The variance in the efficiency of compounds in contradiction ofdiverse organisms depends on either the impenetrability of the cell microorganisms or dispersion in the ribosomes of microbial cells. The results obtained after screening the synthesized compounds against various microorganisms are shown in Table 4.

**Table 4:** Antibacterial and antifungal activity of the synthesized compounds.

Comp.	E. Coli	P. Aeruginosa	S. Aureus	C. Albicans
1	10	8	10	9
2	11	7	12	10
3a	10	9	11	9
3b	10	7	8	8
3c	8	-	9	10
3d	10	8	10	8
3e	10	7	7	10
3f	8	8	8	8
3g	10	7	7	8
3h	8	-	9	8
3i	8	8	10	7
4a	12	10	12	11
4b	10	9	11	9
4c	10	-	10	11
4d	10	9	10	9
4e	10	9	11	12
4f	09	8	11	9
4g	10	9	10	8
4h	09	8	11	10
4i	12	8	9	9
SA	35	13	-	-
SD	18	15	23	-
SF	-	-	-	33

*100 µg/mL: drug concentration; SA: standard Ampicillin; SD: standard doxycycline; SF: standard fluconazole.

## 4. SwissADME pharmacokinetics, physicochemical, and medicinal properties study [30]

**Figure 3:**
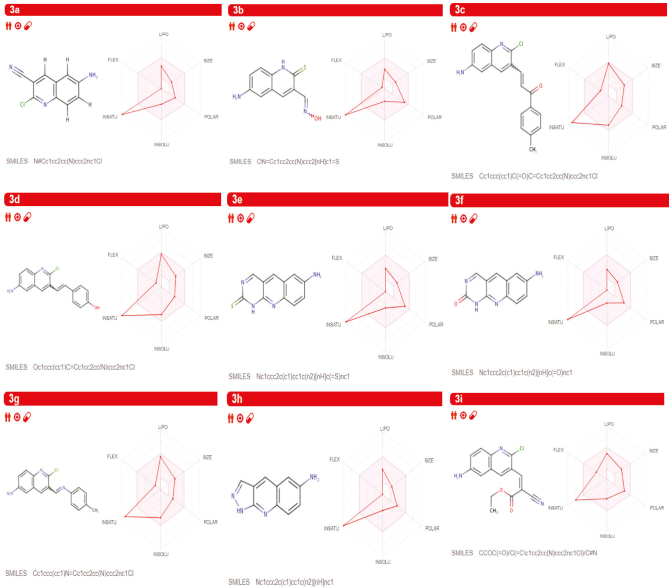
Bioavailability radar of the synthesized molecules 3a–3i evaluating using SwissADME web tool.

The functioning highlights of these molecules were entered into the SwissADME site (http://swissadme.ch) using the ChemAxon’s Marvin JS structure drawing instrument (Budapest, Hungary). Assistant highlights of the pharmacophore sway included bioavailability, transport properties, sympathy to proteins,reactivity, poisonous quality, and metabolic steadiness. Unique to SwissADME is the bioavailability radar [31], which gives a graphical assessment of the prescription resemblance parameters of an orally-accessible bioactive drug. The drug impression chart is exhibited as a hexagon (Figure 3) with every one of the vertices dialogs to a restriction that symbolizes a bioavailable drug.

The pink location inside the hexagon speaks to the perfect range for each property (lipophilicity: XLOGP3 somewhere in the array of −0.7 and +5.0, size: extremity: topological polar surface area (TPSA) somewhere in the field of 20 and 130 Å2, MW somewhere in the scope of 150 g/mol and 500g/mol, solubility: log S not higher than 6, adaptability: close to 9 rotatable bonds, and saturation: some portion of carbons in the sp3 hybridization at any rate 0.25). The recommended run for the compound as bioavailable medication is portrayed in Table 5.

**Table 5:** Physicochemical properties of synthesized molecules 3a–3i.

Sr. No.	MW	HA	AHA	FCsp3	RTB	HBA	HBD	MR	TPSA
3a	203.63	14	10	0	0	2	1	55.87	62.7
3b	219.26	15	10	0	1	2	3	62.92	106.49
3c	322.79	23	16	0.05	3	2	1	95.93	55.98
3d	296.75	21	16	0	2	2	2	88.55	59.14
3e	228.27	16	14	0	0	2	2	66.63	99.68
3f	212.21	16	14	0	0	3	2	62.07	84.66
3g	295.77	21	16	0.06	2	2	1	89.82	51.27
3h	184.2	14	13	0	0	2	2	55.8	67.59
3i	301.73	21	10	0.13	4	4	1	81.51	89

MW: molecular weight, HA: heavy atoms, AHA: aromatic heavy atom, FCsp3: fraction Csp3, RTB: rotatable bonds, HBA: H-bond acceptors, HBD: H-bond donors, MR: molar refractivity, TPSA: topological polar surface area.

### 4.1. Drug-likeness

The drug resemblance properties of the fused compound are indicated by the red mutilated hexagon inside of the pink shade (Figure 3). SwissADME, likewise, has computational channels that incorporate Ghose [32], Egan [33], Veber [34], and Muegee [35], created by top pharmaceutical organizations and cheminfomaticians to assess the drug resemblance of molecules. The values maintain in Table 6 indicated the violation of the prescribed range for the molecule to act as a drug.

**Table 6:** Drug-likeness evaluation of the synthesized compounds using SwissADME.

Sr. No.	Lipinski #violations	Ghose #violations	Veber #violations	Egan #violations	Muegge #violations
3a	0	0	0	0	0
3b	0	0	0	0	0
3c	0	0	0	0	0
3d	0	0	0	0	0
3e	0	0	0	0	0
3f	0	0	0	0	0
3g	0	0	0	0	0
3h	0	0	0	0	1
3i	0	0	0	0	0

### 4.2. PAINS, break, and leadlikeness screening

Pan-assay interference compound (PAINS) screening, which regularly gives bogus positive chemical properties, brings about high-throughput screens. PAINS will in general respond vaguely with various biological targets as opposed to explicitly influencing one foreseen objective. The SwissADME evaluation did not post any PAINS alert (Table 7). In additional to the verdict model, Brenk [36] measured combinations that were smaller and less hydrophobic, and not those described by Lipinski’s standard of 5 to augment open doors for lead streamlining. Leadlikeness tests have been projected to provide primes with extraordinary kinship in high-throughput screens that consider the recognition and control of new exchanges ahead of the pack headway stage (Table 7). All of the synthesized compounds (3a–3i) drifted the break alert and leadlikeness criteria.

**Table 7:** Medicinal chemistry evaluation of the synthesized compounds.

Sr. No.	PAINS #alerts	Brenk #alerts	Leadlikeness #violations	Synthetic accessibility
3a	0	2	1	1.71
3b	0	5	1	2.1
3c	0	3	1	2.88
3d	0	2	1	2.39
3e	0	3	1	1.63
3f	0	2	1	1.55
3g	0	3	1	2.65
3h	0	1	1	1.64
3i	0	4	0	2.76

### 4.3. P-glycoprotein and CYP enzyme activity prediction

SwissADME, furthermore, permits the valuation for a compound to be a substrate of p-glycoprotein (P-gp) or inhibitor of the cytochrome p450 isoenzymes (CYP isoenzymes). P-gp is largely spread and linked in the intestinal epithelium where it drives xenobiotics [37]. The generations return Yes or No if the particle under assessment has a higher probability to be a substrate or nonsubstrate of P-gp or inhibitor or noninhibitor of a given CYP. The screening results were classified in Table 8.

**Table 8:** Pharmacokinetic evaluation of the synthesized compounds.

Sr. No.	GIabs.	BBB per.	P-gp sub.	CYP1A2 inhibitor	CYP2C19 inhibitor	CYP2C9 inhibitor	CYP2D6 inhibitor	CYP3A4 inhibitor
3a	High	Yes	No	Yes	No	No	No	No
3b	High	No	No	Yes	Yes	No	No	No
3c	High	Yes	No	Yes	Yes	Yes	No	Yes
3d	High	Yes	No	Yes	Yes	No	Yes	Yes
3e	High	No	No	Yes	No	No	No	No
3f	High	No	No	Yes	No	No	No	No
3g	High	Yes	No	Yes	Yes	Yes	No	Yes
3h	High	Yes	Yes	Yes	No	No	No	No
3i	High	No	No	Yes	Yes	Yes	No	No

GIabs: gastrointestinal absorption,BBBper: blood-brain barrier permeant, P-gp sub: P-glycoprotein substrate, CYP: cytochromes.

### 4.4. Human intestinal absorption and blood-brain barrier prediction

**Figure 4:**
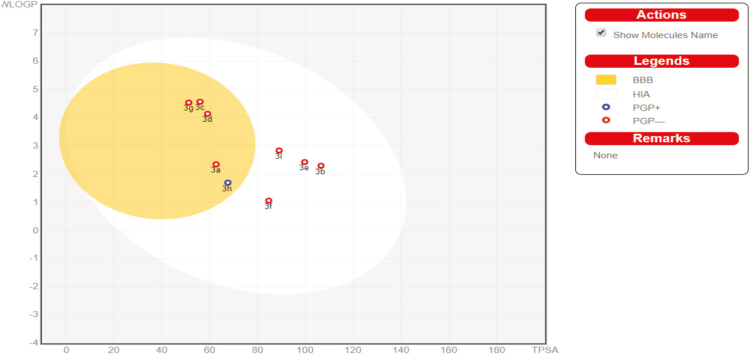
BOILED-Egg allows for evaluation of passive gastrointestinal absorption (HIA), brain penetration (BBB), and P-glycoprotein in the presence of the molecule (P-gp).

Suitable for P-gp and CYP protein vitality is human intestinal absorption (HIA) and blood-brain barrier (BBB) infiltration. SwissADME BOILEDegg (Figure 4) grants the appraisal of HIA as a component of the circumstance of the molecules in the WLOGP-versus-TPSA referential.

The white segment of the BOILEDegg is for a high chance of reflexive adjustment by the gastrointestinal tract, and the yellow region (yolk) is for a high prospect of cerebrum entrance, and it can be seen that some of the blended particles (3b,3e,3f, and 3i) are under the white segment. With this, the focuses are concealed in blue at whatever point was foreseen as adequately effluxed by P-gp (PGP+), shown by 3h and in red, anticipated as a nonsubstrate of P-gp (PGP−) by 3a, 3b, 3c, 3d, and 3g. HIA and the BBB are responsible for the water solvency and lipophilicity of the medication. There are 2 topological ways to deal with the predicted water solvency contained in the SwissADME. The first was an implementation of the estimated solubility (ESOL) [38] model, and the resulting one was improved by Ali et al. [39]. Silicos-IT made SwissADME third pointer for molar solvency. Every single foreseen quality is the decimal logarithm of the molar solvency in water (log S) portrayed in Table 9. Agreement Log p is the normal estimation of all Log P assessed with different lipophilicity criteria (Table 10).

**Table 9:** Water solubility evaluation of the synthesized compounds.

Sr.No.	ESOL				Ali				Silicos			
	Log S	Solu.(mg/mL)	Solu.(mol/L)	Class	Log S	Solu.(mg/mL)	Solu.(mol/L)	Class	Log S	Solu.(mg/mL)	Solu.(mol/L)	Class
3a	–3.01	1.99E-01	9.76E-04	S	–3.14	1.47E-01	7.24E-04	S	–4.05	1.82E-02	8.93E-05	MS
3b	–2.37	9.35E-01	4.26E-03	S	–3.01	2.13E-01	9.73E-04	S	–2.98	2.31E-01	1.05E-03	S
3c	–5.06	2.84E-03	8.79E-06	MS	–5.5	1.02E-03	3.16E-06	MS	–6.88	4.27E-05	1.32E-07	PS
3d	–4.87	3.99E-03	1.35E-05	MS	–5.34	1.36E-03	4.59E-06	MS	–5.98	3.11E-04	1.05E-06	MS
3e	–2.68	4.79E-01	2.10E-03	S	–2.92	2.74E-01	1.20E-03	S	–4.39	9.28E-03	4.06E-05	MS
3f	–2.2	1.34E+00	6.31E-03	S	–1.98	2.21E+00	1.04E-02	VS	–4.19	1.38E-02	6.50E-05	MS
3g	–4.71	5.80E-03	1.96E-05	MS	–4.91	3.61E-03	1.22E-05	MS	–6.95	3.35E-05	1.13E-07	PS
3h	–2.6	4.68E-01	2.54E-03	S	–2.5	5.88E-01	3.19E-03	S	–3.85	2.57E-02	1.40E-04	S
3i	–3.71	5.83E-02	1.93E-04	S	–4.57	8.03E-03	2.66E-05	MS	–4.6	7.51E-03	2.49E-05	MS

Solu.: solubility; PS: poorly soluble; MS: moderately soluble; S: soluble; VS: very soluble.

**Table 10:** Lipophilicity evaluation of the synthesized compounds.

Sr. No.	iLOGP	XLOGP3	WLOGP	MLOGP	Silicos-IT Log P	Consensus Log P
3a	1.5	2.19	2.35	1.09	2.3	1.89
3b	1.4	1.18	2.3	0.41	3.13	1.68
3c	2.81	4.6	4.57	3.05	4.87	3.98
3d	2.48	4.38	4.14	2.93	3.95	3.58
3e	1.41	1.23	2.43	1.12	3.25	1.89
3f	1.03	0.63	1.06	1.11	1.8	1.13
3g	2.66	4.13	4.54	2.93	4.75	3.8
3h	0.98	1.47	1.7	1.35	1.78	1.46
3i	2.44	3.04	2.84	1.39	2.89	2.52

## 5. Structure-activity relationship

**Figure 5:**
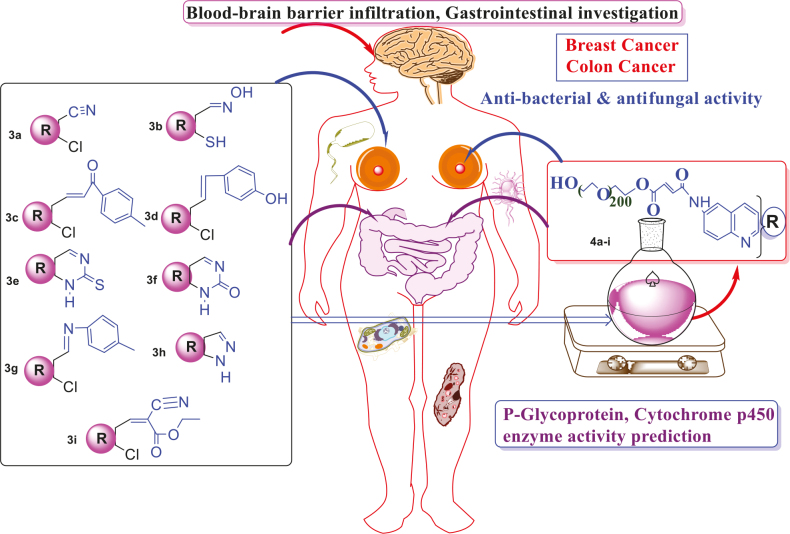
N-PEG quinoline scaffold derivatives.

Depending upon the structural features, the newly synthesized molecules (4a–4i) could be divided into 3 discrete segments, as the diversely substituted central core quinoline ring A PEGylation center, ring B, and substitution at C-2/C-3 (Figure 5).

The exceptionally high anticancer activity of PEGylated quinoline derivatives is suggestive of the fact that the enhanced efficacy of molecules maybe because of the high mobility of the end terminal of the tethered PEG molecules, and due to the presence of inter-repulsive forces exerted between the hydrated PEG chains. There is a strong possibility of single PEG molecule conjugation with the approaching protein.

The data evident in Table 3 led to another noteworthy deduction, that the molecules with unsaturated centers at C-3 position exhibited good to moderate anticancer activity against the cancer cell lines. In contrast, the quinoline scaffold that amalgamated with the fused pyrazole and pyrimidine ring structures showed excellent anticancer activity against the SW620, MD468, and MD468-2 colon cancer cell lines. However, the imine derivatives of the PEGylated quinoline did not display significant activity. These annotations proposed that C-2 and C-3 played a vital role in the structure-activity relationship of compounds 4a–4i.Compounds 4a and 4i were found to be most potent amongst the synthesized compounds against the microbial strains of E. Coli. The rest of these compounds showed moderate to potent activities against S. Aureus. All of the compounds screened against S. Aureus showed moderate to potent activities, confirming the fact that the substituted quinoline, with an adjacent ring C like the cyano group and pyrazole, played a vital role in enhancing the efficacy of the compounds, thus modulating the antibacterial activity.

All of the compounds screened as antifungals showed moderate to potent activities. The synthesized PEGylated quinoline was found to possess moderate antifungal activity against *C. Albicans* .

## 6. Synthesis of 2- chloro-6-nitro-3-formilquinoline (1):[40]

To the solution of p-nitro acetanilide (5 mmol) in dry DMF (15 mmol) at 0–5 °C, POCl3 (35 mmol) was added dropwise and stirred, and then the mixture was stirred at 80–90 °C for 16–19 h. The mixture was poured on to crushed ice, stirred for 5 min, and the resulting solid was filtered, washed well with water, and dried. The compound recrystallized from ethyl acetate and methanol m.p. 260–262 °C. Yield 86%. Color: brownish yellow.

IR (KBr, cm–1); 1685, 1675, 1640.1HNMR (DMSO- *d* 6); δ 7.90–7.91 (1H, q), 7.50–7.53 (1H, d, J= 14.24), 7.70–7.72 (1H, d, J = 8.96), 8.15 (1H, s), 9.74 (1H, s, CHO). 13CNMR (DMSO- *d* 6); δ122.82, 123.25, 124.23, 125.13, 126.91, 142.33, 144.56, 146.33, 153.95, 190.26. MS ( *m/z)* : 236. Elemental analysis: (C10H5ClN2O3): calculated C: 50.76, H: 2.13, Cl: 14.98, N: 11.84, O: 20.29; found: C: 50.64, H: 2.8, Cl: 14.85, N: 11.73, O: 20.18.

### 6.1. Reduction of -NO2 quinoline

**Synthesis of 6-amino-2-chloroquinoline-3-carbaldehyde (2):**

Dissolve sodium sulfide (2 g) in water (5–6 mL) was added to NaHCO3 (0.7 g) in small portions and stirred. When the added NaHCO3had dissolved, methanol (5–6 mL) was added and the reaction mixture was cooled to below 20 °C. Filtering off of the precipitated sodium carbonate was performed with pump. The precipitate was then washed with a small amount of methanol. The filtrate was then used for further reactions.

Next, 2-chloro-6-nitro-3-formilquinoline (0.75 g) was dissolved in hot methanol (6 mL), added to the above solution containing sodium hydrogen sulfide, and shaken. The reaction mixture refluxed for 2 h. The reaction mixture was then allowed to cool and was then placed into ice water (20–40 mL) to filter the reduced compound, and it was then recrystallized from methanol. m.p.208–210 °C. Yield: 82%. Color: dark brown.

IR (KBr, cm–1); 3458, 1675, 1620.1HNMR (DMSO- *d* 6); δ 4.92 (2H, s, -NH _2_ ), 7.52–7.53 (1H, d, J= 7.28), 7.64–7.66 (1H, d, J = 7.56), 7.80–7.84 (1H, q), 8.10 (1H, s), 9.51 (1H, s, CHO). 13CNMR (DMSO- *d* 6); δ122.82, 123.25, 124.23, 125.13, 126.91, 142.33, 144.56, 146.33, 153.95, 190.26. MS ( *m/z)* :206. Elemental analysis: (C10H7ClN2O): calculated C: 58.13, H: 3.41, Cl: 17.16, N: 13.56, O: 7.74; found: C: 58.5, H: 3.30, Cl: 17.11, N: 13.43, O: 7.65.

### 6.2. Synthesis of 6-amino-2-chloroquinoline-3-carbonitrile (3a)

A suspension of 6-amino-2-chloroquinoline-3-carbaldehyde (1 mol) in 30% aq. ammonia was stirred for 15–25 min at room temperature, to this CAN was added at 0–2 °C, andthe reaction mixture was stirred for 2–3 h at 0–2 °C (after completion of the reaction monitored by TLC, the disappearance of the reddish-brown color of the reaction mixture in 30–40 min was observed), extracted with chloroform-ethyl acetate (7:3), dried over Na2SO4,and evaporated under reduced pressure to obtain the solid product of 6-amino-2-chloroquinoline-3-carbonitrile (3a). The solid product was crystallized with ethanol. m.p. 162 °C. Yield: 83%.Color: brown.

IR (KBr, cm–1); 4365, 2115, 1650. 1HNMR (DMSO- *d* 6); δ 4. 68 (2H, s, NH _2_ ), 7.23–7.26 (1H, d, J= 12.4), 7.64 (1H, s), 7.80–7.84 (1H, q), 8.10–8.13 (1H, d, J = 9.56). 13CNMR (DMSO- *d* 6); δ43.57, 105.36, 116.82, 123.25, 129.23, 131.13, 133.51, 138.56, 143.33, 153.95.MS ( *m/z)* :203. Elemental analysis: (C10H6ClN3): calculated C: 58.98, H: 2.97, Cl: 17.41, N: 20.64; found: C: 58.85, H: 2.9, Cl: 17.32, N: 20.58.

### 6.3. Synthesis of 6-amino-3-hydroxyiminomethylquinoline-2(1H)-thione (3b)

To the solution of 6-amino-2-chloro quinoline-3-carbaldehyde (1.5mmol) in dry DMF, fused sodium sulfide was added and stirred for 3–4 h at room temperature. Hydroxylamine hydrochloride and sodium acetate were further added with continued stirring for 4–5 h. On completion, the reaction mixture was poured into ice-cold water and the crude product of 6-amino-3-hydroxyiminomethylquinoline-2(1 *H* )-thione (3b) was achieved, and purified by recrystallization from aq. ethanol. m.p. 168 °C.Yield: 78%. Color: yellow.

IR(KBr, cm–1); 3465, 3455, 2725, 1675, 1215.1HNMR (DMSO- *d* 6); δ3.76 (1H, SH), 4.87 (2H, s, NH _2_ ), aromatic reg.: 6.40–6.80 (4H, m), 7.28 (1H, s, CH=N), 10.58 (1H, s, OH).13CNMR(DMSO- *d* 6); δ113.36, 116.86, 123.35, 125.66, 134.29, 136.95, 145.63, 155.95, 185.45.MS ( *m/z)* :219. Elemental analysis: (C10H9ClN3OS): calculated C: 54.78, H: 4.14, N: 19.16, O: 7.30, S: 14.62; found: C: 54.60, H: 4.8, N: 19.12, O: 7.24, S: 14.53.

### 6.4. Synthesis of 3-(6-amino-2-chloroquinoline-3-yl)-1-p-tolylprop-2-en-1-one (3c)

A mixture of 6-amino-2-chloroquinoline-3-carbaldehyde (0.01 mol, 1.91 g) and4-methyl acetophenone in 40% ethanolic NaOH was stirred vigorously for 2 h and kept overnight at room temperature. The reaction mixture was poured onto crushed ice and acidified with 1:1 HCl. The solid 3-(6-amino-2-chloroquinoline-3-yl)-1- *p* -tolylprop-2-en-1-one (3c) was isolated. It was recrystallized using acetic acid and ethanol. m.p. 240 °C.Yield: 84%. Color: yellowish-white.

IR(KBr, cm–1); 3435, 3015, 2980, 1685.1HNMR (DMSO- *d* 6);δ 2.51 (3H, s, CH3), 5.25 (2H, s, NH _2_ ), 7.24–7.26 (1H, d, J= 9.12), 7.53–7.58 (3H, m), 7.84–7.86 (1H, d, J= 9.16), 8.04 (1H, s), (4H, m, Ben.), 13CNMR(DMSO- *d* 6); δ24.53, 119.15, 123.35, 125.39, 129.15–129.33, 134.16, 136.94, 139.15, 141.13, 144.62, 148.15, 151.59, and 192.16 ppm, MS ( *m/z)* :322. Elemental analysis: (C19H15ClN2O): calculated C: 70.70, H: 4.68, Cl: 10.98, N: 8.68, O: 4.96: found: C: 70.62, H: 4.57, Cl: 10.88, N: 8.58, O: 4.91.

### 6.5. Synthesis of 4(2-(6-amino-2-chloroquinoline-3-yl)vinylphenol (3d)

A mixture of 6-amino-2-chloroquinoline-3-carbaldehyde (0.01 mol), p-cresol (0.01 mol), freshly distilled acetic anhydride (10 mL), acetic acid (10 mL), and sodium acetate (2 g) was heated and refluxed in an oil bath at 145 °C for 6–7 h. After cooling, the reaction mixture was poured onto crushed ice and left overnight. The precipitated product was filtered, dried, and crystallized from ethyl acetate-methanol. m.p. 168 °C. Yield: 83%. Color: brown.

IR(KBr, cm–1);3480, 3425, 3015. 1HNMR (DMSO- *d* 6);δ 3.25 (2H, s, NH _2_ ), 6.53–6.54 (1H, d, J= 3.64), 6.68–6.69 (1H, d, J= 2.00), 7.77–7.83 (2H, m, Ben.), 7.95–7.97 (1H, 1H, d, J= 8.96), 8.12–8.20 (2H, m, Ben.), 8.40–8.43 (1H, d, J= 8.96), 8.68 (1H, s), 10.29 (1H, s, OH). 13CNMR (DMSO- *d* 6); δ118.23, 123.05, 127.51, 129.03, 131.13, 133.51, 135.29, 141.31, 143.33, 148.38, 159.35. MS ( *m/z)* :296. Elemental analysis: (C17H13ClN2O): calculated C: 68.81, H: 4.42, Cl: 11.95, N: 9.44, O: 5.39: found: C: 68.74, H: 4.36, Cl: 11.86, N: 9.37, O: 5.31.

### 6.6. Synthesis of 7-aminopyrimido(4,5-b)quinoline-2(1H)thione (3e)

6-amino-2-chloroquinoline-3-carbaldehyde (0.002 mol) and (0.002 mol) were dissolved in 15 mL of glacial acetic acid and refluxed for 15 h. On completion, the reaction mixture was poured onto crushed ice and neutralized with diluted NaOH. The solid product, 7-aminopyrimido-(4,5-b)-quinoline-2(1 H)thione (3e), was separated out. m.p. 272 °C.Yield: 82%. Color: yellowish-brown.

IR (KBr, cm–1); 3396, 3440, 1655, 1235. 1HNMR(DMSO- *d* 6); δ 5.49 (2H, s, NH _2_ ), 7.83–8.50 (aromatic reg. 5H, m), 11.50 (1H, s, NH).13CNMR (DMSO- *d* 6;115.12, 120.36, 122.15, 129.13, 132.23, 135.55, 142.13, 159.56, 161.59, 182.25. MS ( *m/z)* :228. Elemental analysis: (C11H8N4S): calculated C: 57.88, H: 3.53, N: 24.54, S: 14.05: found: C: 57.79, H: 3.46, N: 24.48, S: 13.95.

### 6.7. Synthesis of 7-aminopyrimido(4,5-b)quinoline-2(1H)one (3f)

6-amino-2-chloroquinoline-3-carbaldehyde (0.002 mol) and urea were dissolved in 15 mL of glacial acetic acid and refluxed for 15 h. On completion (inferred through TLC), the mixture was poured onto crushed ice and neutralized with diluted NaOH. The solid product, 7-aminopyrimido (4, 5-b) quinoline-2(1 H)one (3f), was separated. m.p. 247 °C. Yield: 78%. Color: brownish-yellow.

IR (KBr, cm–1); 3485, 3440, 1735, 1640. 1HNMR (DMSO- *d* 6); δ 6.62 (2H, s, NH _2_ ), 7.46–8.83 (aromatic reg. 5H, m), 11.32(1H, s, NH).13CNMR (DMSO- *d* 6); 115.02, 118.62, 121.81, 128.11, 133.53, 135.65, 149.64, 156.95, 161.55, 181.52. MS ( *m/z)* :212. Elemental analysis: (C11H8N4O): calculated C: 62.26, H: 3.80, N: 26.40, O: 7.54: found: C: 62.18, H: 3.71, N: 26.32, O: 7.48.

### 6.8. Synthesis of 3-(P-tolylimino)methyl-2-chloroquinoline-6-amine (3g)

To the solution of 6-amino-2-chloroquinoline- 3-carbaldehyde (1mol) and p-toluene in 15 mL methanol was added 1 mL of acetic acid. The reaction mixture was refluxed for 5–6 h. Completion of the reaction was inferred through TLC, after which, it was cooled, the precipitate formed, and the product of 3-( *P* -tolylimino)methyl-2-chloroquinoline-6 amine (3g) separated. It was filtered off, dried, and crystallized with ethanol. m.p. 176 °C. Yield: 86%. Color: darkbrown.

IR (KBr, cm–1); 3460, 1650, 1635. 1HNMR (DMSO- *d* 6); δ 2.50 (3H, s, CH3), 5.24 (2H, s, NH _2_ ), 6.06–9.27 (aromatic reg. 9H, m), 13CNMR (DMSO- *d* 6); 24.35, 118.12, 120.05, 122.35, 125.15, 129.03, 131.13, 134.23, 136.95, 142.13, 144.62, 146.15, 148.56, 195.16. MS ( *m/z)* :296. Elemental analysis: (C17H14ClN3): calculated C: 69.03, H: 4.77, Cl: 11.99, N: 14.21: found: C: 68.97, H: 4.69, Cl: 11.91, N: 14.18.

### 6.9. Synthesis of 1H-pyrazolo(3,4-b)quinoli-6-amine (3h)

6-amino-2-chloroquinoline-3-carbaldehyde (0.02 mol) was refluxed with 5 mL (excess) of NH _2_ -NH _2_ in ethanol for 5–6h. Their action mixture was then cooled and poured into ice-cold water. The solid that formed was filtered, washed with water, and dried. It was crystallized with ethyl acetate. m.p. 288 °C.Yield: 76%. Color: yellowish-brown.

IR (KBr, cm–1); 3475, 3318–3325, 1650. 1HNMR (DMSO- *d* 6); δ 3.87 (2H, s, NH _2_ ), 6.32 (1H, s), 6.87 (1H, s), 6.60–6.62 (2H, m), 7.93 (1H, s), 10.43 (1H, s, NH). 13CNMR (DMSO- *d* 6); 120.13–120.32, 122.25, 130.31–131.23, 132.95, 135.13, 139.23–139.59, 141.11, 142.55, 154.13, 182.25.MS ( *m/z)* :184. Elemental analysis: (C10H8N3): calculated C: 65.21, H: 4.38, N: 30.40: found: C: 65.13, H: 4.28, N: 30.31.

### 6.10. Synthesis of (Z)-ethyl-3-(6-amino-2-chloroquinoline-3-yl)-2-cyanoacrylate (3i)

To the solution of 6-amino-2-chloroquinoline-3-carbaldehyde (0.005 mol) and ethyl cyanoacetate in 30 mL ethanol, triethylamine was added. The reaction mixture was refluxed for 3 h. On completion of the reaction, the solution was poured into ice-cold water, and a solid product of (Z)-ethyl-3-(6-amino-2-chloroquinoline-3-yl)-2-cyanoacrylate (3i) was separated. It was washed with cold water and dried, and then recrystallized with methanol. m.p.122 °C. Yield: 86%. Color: white.

IR(KBr, cm–1); 3505, 3022, 2985, 2115, 1720.1HNMR(DMSO- *d* 6); δ 1.21–1.38 (3H, m, CH3), 3.09–3.14 (2H, m, CH _2_ ), 4.20 (2H, s, NH _2_ ), 6.34 (1H, s, CH=C), 6.59–8.40 (aromatic reg. 4H, m), 13CNMR (DMSO- *d* 6);18.51, 96.24, 114.24, 120.51, 128.11, 130.23, 132.85, 141.53, 143.35, 149.64, 156.91, 168.52. MS ( *m/z)* :302. Elemental analysis: (C15H12ClN3O2): calculated C: 59.71, H: 4.01, Cl: 11.75, N: 13.93, O: 10.61: found: C: 59.63, H: 3.93, Cl: 11.68, N: 13.85, O: 10.54.

## 7. Synthesis of hydroxy carboxy polyethylene glycol (HO-PEG200COOH)

Polyethylene glycol (5 mL, 0.028 mol, 200 g/mol) was dissolved in 20 mL of dry CH _2_ Cl _2_ . To this solution was added THF containing maleic anhydride (0.056 mol, 0.54 mg) and pyridine (0.056 mol, 0.46 mL). After stirring overnight at room temperature, the reaction was completed, as indicated by TLC (methanol:ethyl acetate, 7:3) (no anhydride was left and there was the detection of a single polar PEG compound). The monoacid derivative of (polyethylene glycol)200 was used without purification.

### 7.1. Synthesis of resin via N-terminal of PEGylated-6-amino-2-chloroquinoline-3-carbonitrile (4a) like syrupy material

The monoacid derivative of hydroxy carboxy polyethylene glycol (HO-PEG200COOH) (0.028 mol) was activated with a 1:2 molar equivalent of 6-amino-2-chloroquinoline-3-carbonitrile (3a) (0.046 mol) and N, N-dicyclocarbidiimide (0.046 mol) was dissolved in dichloromethane. The solution was stirred over night at room temperature. This reaction mixture appeared like a resin when dried under a vacuum. This syrupy resin was dissolved in 15 mL of acetone. Later the white precipitate of DCU that appeared was discarded and the filtrate was collected. The final filtrate was evaporated to afford compound 4a. TLC in (methanol:ethyl acetate 7:3) was performed to check for the presence of DCU. It showed a negative result. The IR spectra confirmed this negative result. A negative dye test indicated complete amino group capping.

The resin was dried in a vacuum for IR, H1NMR, C13NMR, and mass spectral analysis. At this stage, the resin did not stick to the glass wall anymore. The IR spectrum of the resin showed the characteristic absorption band of the PEG ether backbone (1120 cm–1) and absorption bands at 1760 and 1685 cm–1 for the ester and amid bond, respectively (C=O).

Color: yellow syrupy material, IR (KBr, cm–1); 3475, 2115, 1685.1HNMR (DMSO- *d* 6);δ 3.04–3.67 (m, PEG)δ 4.72 (1H, s, -NH), 6.56 (1H, s),6.85–6.87 (1H, q), 7.25 (1H, s),7.50–7.52 (1H, d, J= 8.36), 13CNMR (DMSO- *d* 6); δ47.57, 55.11, 55.53, 63.52, 68.53, 107.80, 116.12, 123.59, 126.33, 131.16, 132.26, 138.65, 143.36, and 149.53: MS ( *m/z)* :485.

### 7.2. Synthesis of PEGylated-6-amino-3-hydroxyiminomethylquinoline-2(1H)-thione (4b).

Color: dark yellow syrupy material, IR (KBr, cm–1); 3440, 2735, 1655, 1230.1HNMR (DMSO- *d* 6);δ 3.49–3.76 (m, PEG), δ 4.01 (1H, s -SH), 5.27 (1H, s, -NH), 6.25(1H, s), 6.63 (1H, s),7.25–7.28 (1H, q), 8.56 (1H, s),8.042–8.047 (1H, d, J= 2.08):13CNMR (DMSO- *d* 6); δ41.37, 46.23–46.57, 54.23–55.33, 61.21, 64.55, 112.12, 116.36, 123.11, 125.13, 134.45, 136.21, 136.25, 145.13, 152.24, and 185.51: MS ( *m/z)* :497.

### 7.3. Synthesis of PEGylated-3-(6-amino-2-chloroquinoline-3-yl)-1-p-tolylprop-2-en-1-one (4c)

Color: yellowish-white syrupy material, IR (KBr, cm–1); 3025. 2965. 1655.1HNMR (DMSO- *d* 6);δ 2.22 (3H, s, -CH3), 3.52–4.33 (m, PEG), δ 5.29 (1H, s, -NH), 6.59–6.61 (1H, d, J= 9.72), 6.82 (2H, s), 7.28–7.31 (4H, m, Ben.), 7.67–7.71 (2H, m), 8.01–8.03 (1H, d, J= 10.36). 13CNMR (DMSO- *d* 6); δ28.34, 45.65, 46.93–50.54, 53.39, 62.55, 119.13, 124.35, 126.43, 129.15–129.63, 143.11, 136.90, 138.55, 140.13, 144.32, 148.15, 153.21, and 190.41: MS ( *m/z)* :602.

### 7.4. Synthesis of PEGylated-4(2-(6-amino-2-chloroquinoline-3-yl)vinyl phenol (4d)

Color: yellowish-brown syrupy material, IR (KBr, cm–1); 3465, 3020, 1635.1HNMR (DMSO- *d* 6); δ 3.31–4.42 (m, PEG), δ 5.27 (1H, s, -NH), 6.26 (1H, s), 6.70 (1H, s), 7.28–7.31 (2H, m), 7.66–7.78(4H, m, Ben.), 8.57–8.59 (1H, d, J= 10.48),8.11–8.13 (1H, d, J= 9.12), 10.67 (1H, s, -OH).13CNMR (DMSO- *d* 6); δ25.51, 29.93, 33.77, 41.03–45.90, 46.37, 118.73, 121.15, 127.51, 129.03, 131.13, 134.57, 137.22, 140.13, 142.39, 153.81, and 159.45: MS ( *m/z)* :576.

### 7.5. Synthesis of PEGylated-7-aminopyrimido(4,5-b)quinoline-2(1 H)thione (4e)

Color: brownish-yellow syrupy material, IR (KBr, cm–1); 3425, 1665, 1245.1HNMR (DMSO- *d* 6); δ 3.04–3.67 (m, PEG), 4.72 (1H, s, -NH), 6.26 (1H, s), 6.73–6.78 (2H, m), 7.26 (1H, s), 7.57 (1H, s), 10.67 (1H, s, -OH). 13CNMR (DMSO- *d* 6); δ29.65, 32.61–36.45, 42.93, 46.55, 115.62, 120.61, 122.52, 129.33, 132.93, 136.55, 142.43, 159.65, 164.49, and 185.15: MS ( *m/z)* :544.

### 7.6. Synthesis of PEGylated-7-aminopyrimido(4,5-b)quinoline-2(1 H)one (4f)

Color: brownish-yellow syrupy material, IR (KBr, cm–1); 3420, 1720, 1650. 1HNMR (DMSO- *d* 6); δ 3.04–3.65 (m, PEG), 5.25 (1H, s, -NH), 6.51 (1H, s), 6.78–6.78 (2H, q),7.12 (1H, s), 7.40 (1H, s), 10.26 (1H, s, -OH). 13CNMR (DMSO- *d* 6); δ28.68, 32.63–36.48, 44.35, 46.85, 114.32, 120.61, 122.50, 128.13, 132.53, 146.93, 195.65, 161.51, and 181.52: MS ( *m/z)* :528.

### 7.7. Synthesis of PEGylated-3-((P-tolylimino)methyl-2-chloroquinoline-6-amine (4g)

Color: brownish-yellow syrupy material, IR (KBr, cm–1); 1655, 1640.1HNMR (DMSO- *d* 6); δ 2.09 (3H, s, -CH3), 3.51–4.27 (m, PEG), 4.81 (1H, s, -NH), 6.98–7.49 (9H, m, aromatic reg.). 13CNMR (DMSO- *d* 6); δ24.35, 35.34, 39.27, 44.03–49.90, 55.43, 116.26, 120.65, 122.35, 126.15, 129.63, 131.19, 136.23, 144.69, 146.42, 148.61, and 159.11: MS ( *m/z)* :606.

### 7.8. Synthesis of PEGylated-1H-pyrazolo(3,4-b)quinoli-6-amine (4h)

Color: brownish-yellow syrupy material, IR (KBr, cm–1); 3445, 3325, 1665.1HNMR (DMSO- *d* 6); δ 3.42–4.31 (m, PEG), 5.28 (1H, s, -NH),6.27 (1H, s) 6.59–8.02 (4H, m, aromatic reg.), 10.31 (1H, s, -NH). 13CNMR (DMSO- *d* 6); δ29.91, 36.65, 39.91–43.45, 49.39, 52.54, 120.31–120.39, 122.52, 130.35–131.33, 132.91, 135.53, 139.32–139.55, 141.18, 142.55, 156.53, and 182.25: MS ( *m/z)* :463.

### 7.9. Synthesis of PEGylated-(Z)-ethyl 3-(6-amino-2-chloroquinoline-3-yl)-2-cyanoacrylate (4i)

Color: yellow syrupy material, IR (KBr, cm–1); 3025, 2975, 2130, 1690.1HNMR (DMSO- *d* 6);δ 1.68 (3H, -CH3), 3.07 (2H, -CH _2_ ), 3.52–4.33 (m, PEG), δ 5.29 (1H, s, -NH), 6.82 (1H, s, CH=C),7.28–8.03 (4H, m, aromatic reg.). 13CNMR (DMSO- *d* 6); δ18.15, 34.36, 36.54–40.35, 43.85, 46.50, 55.98, 90.85, 114.42, 120.21, 128.12, 139.23, 136.89, 141.53, 143.38, 151.64, 154.45, and 168.52: MS ( *m/z)* :581.
